# Non-Pharmacological Approaches for Migraine

**DOI:** 10.1007/s13311-018-0623-6

**Published:** 2018-04-03

**Authors:** Francesca Puledda, Kevin Shields

**Affiliations:** 1 Department of Basic and Clinical Neuroscience, Institute of Psychiatry, Psychology and Neuroscience, King’s College London, London, UK; 2 Headache Service, The National Hospital for Neurology and Neurosurgery, Queen Square, London, UK

**Keywords:** Migraine, Therapy, Non-pharmacological, Neuromodulation, Nutraceuticals

## Abstract

Migraine is one of the most common and debilitating neurological disorders. However, the efficacy of pharmacological therapies may have unsatisfactory efficacy and can be poorly tolerated. There is a strong need in clinical practice for alternative approaches for both acute and preventive treatment. Occasionally, this need might arise in the context of low-frequency migraneurs who are not keen to use medication or fear the potential side effects. At the opposite end of the spectrum, clinicians might be faced with patients who have proven refractory to numerous medications. These patients may benefit from invasive treatment strategies. In recent years, promising strategies for migraine therapy have emerged alongside a progressively better understanding of the complex pathophysiology underlying this disease. This review discusses the most recent and evidence-based advances in non-pharmacological therapeutic approaches for migraine, offering alternatives to drug treatment for both the commonly encountered episodic cases as well as the more complex migraine phenotypes, which are capable of challenging even the headache specialist.

**Electronic supplementary material:**

The online version of this article (10.1007/s13311-018-0623-6) contains supplementary material, which is available to authorized users.

## Introduction

Migraine is one of the most common neurological diseases with a possible cumulative lifetime incidence of up to 50% in women and 20% in men [1]. Its high prevalence and frequency in young people at the peak of their productive years causes it to rank as the sixth cause of disability in the world [2].

The currently available oral pharmacological migraine treatments may be poorly tolerated by some patients. Unpleasant side effects and lower than hoped for efficacy [3, 4] can lead to low treatment compliance and other complications, such as headache chronification and acute medication overuse. However, recent years have seen a proliferation in new therapeutic strategies for tackling migraine, as well as novel techniques that offer promising areas of development. This review will focus on acute and preventive non-pharmacological approaches to migraine therapy. We will particularly concentrate on new strategies backed by robust evidence, prioritizing the attention of the reader on randomized controlled trials (RCTs), when these are available.

The treatments that are analyzed in this article range from commonly used and easily accessible nutraceuticals, like magnesium and riboflavin, to well-established psychological therapies such as cognitive-behavioral therapy (CBT). Finally, we will focus on the relatively novel area of neuromodulation. With respect to the latter, we will analyze in detail the available non-invasive techniques (the most significant of which are summarized in Table 1). These may potentially offer an alternative for episodic migraine patients who hope to avoid the side effects of pharmacological therapies. We will also briefly review the invasive strategies—represented by stimulation of the occipital nerve, the sphenopalatine ganglion, or the cervical spinal cord—which can be considered in chronic migraine patients who have repeatedly failed previous approaches.

**Table 1:**
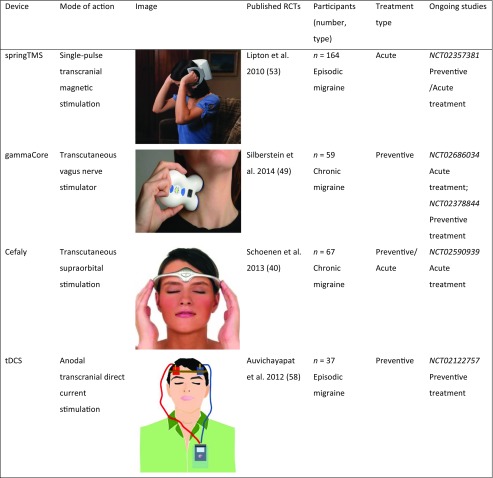
Main randomized controlled trials for non-invasive neuromodulation in migraine

The importance in clinical practice of being able to offer patients who struggle with classic drug treatments relatively low risk and well-tolerated alternatives is obvious. Furthermore, it is of particular interest for the headache specialist to become familiar with the expanding world of neuromodulation, which aside from allowing strategies that bypass regular medication side effects, may offer insight into pathophysiological mechanisms of migraine and its underlying biology.

## Nutraceuticals

Nutraceuticals are defined as food or dietary supplements that provide medicinal or health benefits. Their use is becoming increasingly popular in the general population. They have a particular appeal to patients with chronic diseases who hope to avoid the issues associated with long-term prescription treatments. In patients with migraine, the use of this kind of non-pharmacological therapy is growing and is likely to be widely underestimated [5].

The most commonly used nutraceuticals which have shown some evidence in migraine prevention are riboflavin (vitamin B2), coenzyme Q10 (CoQ10), magnesium, butterbur root extract (*Petasites hybridus*), and feverfew (*Tanacetum parthenium*).

Riboflavin is a precursor of flavin mononucleotide and flavin adenine dinucleotide. These coenzymes are required for several energy-related cellular functions and electron transport within the Krebs cycle, and so play an important role in energy production inside the mitochondrion. The rationale for using riboflavin in migraine emanated from magnetic resonance spectroscopy studies [6–8]. These suggested that there may be mitochondrial dysfunction within the migranous brain. To date, five randomized controlled trials have assessed the efficacy of riboflavin in migraine, with somewhat conflicting results. The first randomized controlled trial assessing the use of riboflavin in migraine was performed in Belgium [9]. In this study, the effect of 400 mg daily dose was tested in 55 episodic adult migraineurs, with and without aura. Riboflavin showed a significant effect in reducing the number of headache days and attack frequency, with only minor and rare side effects when compared with placebo. However, a randomized control trial on 48 children with episodic migraine failed to show any difference between a 50% lower dose of 200 mg riboflavin and placebo in reducing the number of headache days, their duration, or the severity of migraine attacks and associated symptoms [10]. These results are in contrast with the study by Athaillah et al. [11] who performed a randomized controlled trial on 98 adolescents between the ages of 12 and 19. In this study, episodic migraineurs both with and without aura were randomly assigned to receive either placebo or riboflavin 400 mg. Treatment subjects in the active group showed a significant reduction in number of migraine days per month, starting from the second month of treatment. Two trials have also tested the effect of riboflavin in combination with other supplements in migraine, with opposite outcomes. In a study by Maizels et al. [12], subjects were randomized to receive either a combination of riboflavin 400 mg, magnesium 300 mg and feverfew 100 or 25 mg of riboflavin daily. This was done in order to achieve similar levels of chromaturia (a typical side effect of riboflavin) in both the active and “placebo” groups to avoid unblinding. Results showed no observed difference in the two groups with regard to reduction in migraine frequency. Given the high placebo response rate seen in the study, it has been speculated by the authors that 25 mg riboflavin might have actually acted as an active compound, therefore confounding comparison. It should also be noted that up to one third of patients in the study were taking concomitant preventive treatments for migraine. In 2015, a large German multicenter study by Gaul et al. [13] compared a compound containing riboflavin 400 mg, coenzyme Q10 150 mg, magnesium 600 mg and multivitamins with placebo in 130 episodic migraneurs. The active group failed to show a significant reduction in number of migraine days with respect to the placebo group; secondary outcome measures such as pain intensity and burden of disease were however significantly reduced. Methodological differences make it difficult to draw definitive conclusions regarding the efficacy of riboflavin. There does appear to be sufficient evidence to recommend that it be used as a well-tolerated, low risk preventive treatment for adults with migraine. Intriguingly there is some evidence to suggest that variations in mitochondrial DNA may influence the response to riboflavin [14]. This might help explain some of the observed variability in efficacy but regrettably it does not allow tailoring of treatment to likely responders.

Similarly to riboflavin, coenzyme Q10 has an essential role in the mitochondrial electron transport chain and energy metabolism. In migraine, it has shown efficacy in one randomized controlled trial to date in which 42 migraine patients received either placebo or a 300-mg daily dose of CoQ10 [15]. The treatment group showed a more pronounced (*p* = 0.05) reduction in attack frequency from baseline to month 4 with respect to placebo, and the product was generally well tolerated. In another RCT on 50 migraine children and adolescents, CoQ10 was given at the dose of 100 mg per day and compared to placebo, showing no difference between the two groups [16]. More evidence will be needed therefore to support the widespread use of CoQ10 as a preventive treatment.

Previous studies have highlighted a reduction of magnesium levels in migraine; these observations lead to the hypothesis of a consequent neuronal hyperexcitability in the migranous brain, given the role of this compound in inhibiting glutamate expression through NMDA receptor binding [17–21]. Oral magnesium has been systematically studied as a preventive treatment for migraine in five randomized controlled trials. The first study, performed by Facchinetti et al., assessed the effect of 360 mg oral magnesium in menstrual migraine compared to placebo [22] in 20 subjects. Both the treatment and placebo group had a significant decrease in migraine frequencies and pain scores with respect to baseline; therefore, magnesium did not prove more effective than placebo with regard to these outcome measures. However, the active group showed significantly reduced pain scores respect to placebo after treatment. Limitations of this study include its small case number and the fact that it was only conducted in women. In a multicenter German study carried out in 43 migraineurs with or without aura, a daily dose of 600 mg magnesium was compared to placebo [23]. The study was designed as a crossover trial in which the 2-month treatment was interchanged. Interestingly, a significant reduction in attack frequency was demonstrated only in the placebo-verum sequence and not in the verum-placebo one, possibly because of a carry-over effect of magnesium due to the absence of a wash-out period in between treatments [24]. Pfaffenrath and colleagues [25] compared magnesium 486 mg daily to placebo in a study involving 69 migraine without aura patients, finding no statistically significant difference in headache days. On the other hand, Peikert et al. [26] compared magnesium 600 mg to placebo in 68 total migraineurs and found significantly decreased attack frequency, duration and intensity in the active group. Finally, a study by Koseoglu on 40 migraineurs without aura showed a significant decrease in attack frequency in the group treated with 600 mg daily magnesium, although a reduction could also be seen in the placebo group [27]. This study however had a net disproportion between group sizes, with 30 patients receiving magnesium and 10 placebo. In conclusion, there is limited evidence to suggest that magnesium constitutes an effective preventive treatment for migraine patients.

*Petasites hybridus* or butterbur root is a herbal extract that has shown some efficacy in migraine prevention. Its name derives from the leaves of the plant, which due to their size were originally used to wrap butter. The extract of the butterbur root which is used in tablet form is called Petadolex and is manufactured in Germany. There have been some safety concerns related to possible liver toxicity with the use of butterbur in recent years [28]. In total, two placebo-controlled trials have been performed in migraine to date. Lipton et al. [29] compared the efficacy of butterbur 50 and 75 mg twice daily with placebo. A significant reduction in migraine attack frequency was seen with the 75 mg dose, with no effect for the lower dose. Diener performed an analysis of a previous RCT [30], showing a significant decrease of migraine attack frequency after treatment with butterbur 100 mg total daily dose compared to placebo after 12 weeks of treatment. Butterbur therefore appears to be effective but safety concerns mean that it cannot currently be recommended as a preventive treatment.

*Tanacetum parthenium* or feverfew has been studied in migraine prophylaxis, although results are still unconvincing and have low quality of evidence. One large RCT [31], conducted on 170 migraine patients, showed overall good tolerability and a reduction in migraine attacks with 6.25 mg of feverfew extract. A previous study using the same extract however had failed to show a significant effect in migraine prophylaxis [32].

## Behavioral Techniques and Acupuncture

Behavioral techniques comprise a series of strategies—relaxation, thermal and electromyographic biofeedback and cognitive behavioral therapy—which have been used in migraine therapy mostly with the aim of teaching patients to better cope with symptoms and identifying potential triggers for headache. Relaxation techniques include progressive muscle relaxation, autogenic training and meditation. Biofeedback training uses electronic devices to help the patient understand and monitor certain physiological processes associated with the experience of pain, such as muscle tension, blood pressure and heart rate changes. Cognitive behavioral therapy is a form of brief and symptom-oriented psychotherapy focused on managing stress. These techniques have some degree of evidence for their use in migraine [33, 34], particularly when there is low tolerance to medical strategies and in specific cases when medication is not indicated, such as pregnancy, medical comorbidities or evidence of previous medication overuse [35]. Additionally, they can be effective in conjunction with classic pharmacological therapies [36, 37] and allow a certain degree of self-management, which can be advantageous for some patients. However, one must consider that these strategies do not target the migraine biology or the actual pain mechanisms.

A recent randomized trial [37] compared cognitive behavioral therapy plus amitriptyline to headache education plus amitriptyline in 135 children and adolescents with chronic migraine. The active CBT therapy group showed a significant reduction in headache days of 11.5 respect to the control group at 20 weeks. Another study on 61 transformed migraine and medication overuse headache patients showed that biofeedback-assisted relaxation combined with pharmacological therapy was more effective in reducing headache days and reduced consumption of analgesic than pharmacological therapy alone [38]; these positive results were confirmed by a more recent pilot randomized study on similar patient groups [39].

The use of acupuncture in migraine has yielded conflicting results. There have been a few RCTs [40] in which acupuncture is compared with either sham acupuncture or standard of care, showing some effect in headache improvement. Nonetheless blinding proves difficult in these studies and overall the strength of the evidence is low. One recent trial showed some minor effect over sham acupuncture [41] albeit with a high likelihood of unblinding, with others showing no difference [42]. The largest RCT investigating the effects of acupuncture in migraine was performed in Germany, on a total of 960 patients (*n* = 794 in the intention-to-treat population) [43]. This study compared the effect of verum acupuncture to sham acupuncture and standard therapy, showing that all three treatments were effective in reducing the number of migraine days respect to baseline, but that there was no significant difference in between the three groups. This allows to speculate that the biological effect of acupuncture might not depend on the positioning of the needles themselves.

## Non-Invasive Neuromodulation

Non-invasive neuromodulation is a burgeoning field in migraine research and treatment. The classic techniques act by stimulating the nervous system centrally or at the periphery; this can be done through the skin either with and electric current or with a fluctuating magnetic field, ultimately modulating pain mechanisms involved in headache. Both modalities can have immediate effects, making them suitable for acute symptomatic treatment, while chronic administration may have longer-term preventive actions. These devices show potential as they offer an alternative to oral or invasive therapies while also having favorable side effect profiles; however, caution is required. As we shall see the randomized placebo-controlled trials published to date have tended to be small, and questions have been raised regarding the degree of blinding. It is therefore difficult to give definitive guidance currently on the efficacy of these treatments.

### Transcutaneous Cranial Nerve Stimulation

Supraorbital nerve stimulation (STS) with the Cefaly® device (Cefaly Technology, Grâce-Hollogne Belgium) is a form of transcutaneous cranial nerve stimulation. It was first studied as a preventive treatment in a pilot study on episodic migraine, resulting in an average reduction of 1.3 headache days in ten participants [44]. This study was followed by an RCT on 67 migraine subjects. Sham stimulation in this trial was obtained with an identical looking and sounding machine. The impulse of the sham stimulation however was of 1 mA intensity and 1 Hz frequency (respectively 16 mA and 60 Hz for verum) and could not be perceived by the subjects. The results of the study showed that a once daily treatment session with Cefaly® for 3 months caused a significant 30% reduction in migraine days. This result was more evident in the active group, even though the difference between the two groups was just above statistical significance. The verum stimulation group also showed a greater 50% responder rate than the sham (38.2 vs 12.1%) with a 26% therapeutic gain [45]. A post marketing survey of 2313 patients who rented the Cefaly® device for 40 days showed that 54% of users thought that the device was beneficial and were satisfied with it [46]. The most frequently reported side effect was paresthesia in the area of stimulation. This side effect, albeit mild and fully reversible, can be intolerable in some and can lead to treatment interruption. It may also have caused a certain degree of unblinding during the randomized controlled trial, but it is still reasonable to consider Cefaly® as a preventive treatment.

An RCT on the use of Cefaly® in the acute treatment of migraine (NCT02590939) and an open-label study in chronic migraine (NCT02342743) have recently been completed and results are awaited. The device is currently available for purchase in Europe, North America and Australia.

Transcutaneous occipital nerve stimulation (tONS) has been trialed for migraine prevention in a recent randomized controlled trial [47]. A HANS TENS machine delivering three different frequencies (2 Hz, 100 Hz, 2/100 Hz) over the occipital area was compared with sham stimulation and 100 mg topiramate daily in 110 subjects. A significant decrease in headache frequencies was observed in the medication group and the 100 Hz stimulation group compared to the sham group, showing a potential for this new strategy in migraine. The Cefaly® device has also been recently used as a transcutaneous occipital nerve stimulator for the treatment of chronic migraine [48], with promising results. Twenty-three patients treated with the device over 3 months had a 17% decrease in headache days and 22% in migraine days; furthermore, abnormal VEP habituation reversed to an episodic migraine pattern in these subjects.

Finally, one randomized controlled trial combined occipital and supraorbital transcutaneous nerve stimulation with an OSTNS Neurostimulator (NCT02438553) for acute migraine relief; results of this study are awaited.

### Non-Invasive Vagus Nerve Stimulation (nVNS)

The gammaCore® device (electroCore, LLC; Basking Ridge, NJ, USA) is a handheld electrical stimulator that delivers a transcutaneous current to the cervical branch of the vagus nerve. Initially, vagus nerve stimulation (VNS) was an invasive procedure used in the treatment of refractory epilepsy and depression. Anecdotal observations of migraine improvement in patients being treated with implantable devices [49], spurred interest in using VNS to treat headache disorders. This leads to the development of the handheld non-invasive gammaCore® device.

The first open-label single-arm study examining the efficacy of gammaCore® for the acute treatment of migraine was performed on 30 (27 in the full analysis set) USA patients, showing a pain free rate at 2 h of 21% [50]. Similar results were achieved in the open-label study by Barbanti and colleagues [51], which showed 2-h pain free rates of 23% in 48 high-frequency episodic and chronic migraine patients. The side effect profile in both studies showed a high tolerability for the device, with the most common adverse events being twitching and a tingling sensation at the stimulation site.

Following these results, nVNS was studied initially with two open label studies as a preventive treatment for migraine. Magis et al. [52] performed a small study on 12 migraine subjects which showed a high improvement, particularly in one patient with medication overuse. However, a high percentage of subjects did stop treatment. More recently, one study in menstrual related migraine showed that a 12-week treatment period of non-invasive vagus nerve stimulation, started 3 days before the estimated onset of menses, was effective in reducing menstrually related migraine of 2.5 days per month, with more than one third of subjects showing a 50% reduction rate [53].

Unfortunately, the only randomized controlled study to test the efficacy of nVNS in migraine prevention to date—the EVENT study [54]—has failed to show a significant difference after 2 months of treatment in the active vs. sham group. Treatment was administered three times a day unilaterally with two 90 s doses in 59 chronic migraine patients. The trial consisted of a randomized phase lasting 2 months followed by an open label phase of 6 months. It is interesting to note, however, that at the end of the open label treatment, participants who had been assigned to nVNS during the randomized phase and therefore completed 8 months of treatment, had a significant reduction of almost eight headache days per month. nVNS therefore has shown some promise as an acute treatment but evidence is lacking to support its use as a preventive therapy. Currently two RCTs are studying gammaCore® both as an acute (NCT02686034) and preventive (NCT02378844) strategy in migraine and their results will be eagerly awaited.

The Nemos® device (Cerbomed, Erlangen, Germany) is a recently developed transcutaneous stimulator of the aurical branch of the vagus nerve. The electrode is worn in the ear. In a German-based randomized controlled trial on 46 subjects, patients were randomly assigned to receive either 25 Hz or 1 Hz stimulation, the latter being intended as a sham stimulation [55]. Patients were asked to stimulate for a total of 4 h per day, in sessions of 1 to 4 h. Interestingly, patients in the “sham” treatment group had a more significant reduction in headache days than the ones in the “active” stimulation group. The response to sham stimulation may possibly indicate some biological activity but there is at present insufficient data to support its regular use in clinical practice. Treatment-related side effects in this study were mostly characterized by local pain, paresthesia and ulcers at the stimulation site.

### Single-Pulse Transcranial Magnetic Stimulation (sTMS)

Single-pulse transcranial magnetic stimulation is a safe and non-invasive technique that has been used in the field of neuroscience for decades. It acts by creating a fluctuating magnetic field which in turn induces an electric current capable of modifying the excitability of cortical neurons and thalamocortical circuits. Animal studies have shown that sTMS is also capable of inhibiting cortical spreading depression, as well as the firing of nociceptive thalamic neurons projecting to the cortex [56], thus justifying the growing interest for this technique in migraine therapy.

The first open-label study of sTMS in migraine was performed on 42 subjects who treated acute migraine attacks with brief pulses of TMS [57]. The encouraging efficacy and safety results of this study lead to the development of a large randomized control trial performed in the USA using a hand-held transcranial magnetic stimulator for the treatment of acute migraine with aura. A total of 164 patients treated at least one aura episode with two sTMS pulses or sham. The 2-h pain free rates were significantly higher in the group using sTMS (39%) compared to the sham group (22%) [58].

A UK open-label study [59] was performed to review the post-market use of SpringTMS ®device (eNeura, Baltimore, USA) in a large number of episodic and chronic migraineurs, both with and without aura. A total of 190 patients, using the device for acute and preventive purposes, reported an overall 60% pain relief rate as well as significant reduction of headache days after 3 months of continuous treatment. An economic comparison between sTMS and Botox® treatment in chronic migraine in the UK also suggests that sTMS offers a cost effective treatment for this group of patients [60].

The ESPOUSE trial (NCT02357381) is an ongoing, USA-based post-marketing study for sTMS treatment in the acute and preventive setting. Initial reports showed similar data to the UK post market survey, with a reduction of three and eight headache days per month, respectively, in episodic and chronic migraine patients.

### Transcranial Direct Current Stimulation (tDCS)

Transcranial direct current stimulation may modulate cortical excitability by an anodal (excitatory) or cathodal (inhibitory) electric current applied to the scalp. This modifies the membrane potential of underlying cortical neurons. The effect of tDCS in migraine prevention has been tested using both anodal and cathodal currents.

The two studies testing cathodal inhibitory currents to date have failed to show an effect of this technique in preventive migraine treatment. The first was a randomized controlled study on 26 migraine patients in which the active electrode was placed on the visual cortex. The study showed no significant reduction in migraine attacks and no difference between the two groups [61]. A smaller, more recent pilot study performed on 15 migraineurs similarly showed no significant difference in number of attacks, pain intensity and duration between sham stimulation and inhibitory tDCS over the area corresponding to the visual cortex [62].

Opposite outcomes seem to be achieved when applying an anodal activating current to the scalp with tDCS. In 2012, Auvichayapat et al. performed a randomized controlled trial on 37 episodic migraine patients in which a 20 min 1 mA anodal current over the primary motor cortex was compared with sham treatment [63]. Results showed a significant reduction in attack frequency and pain intensity in the active stimulation group. This result however was only seen at 4 and 8 weeks after treatment and not at 12 weeks, suggesting a possible short-term effect. Using a similar anatomical approach and a 2 mA current, DaSilva et al. also showed a significant reduction in headache intensity and a trend of reduced frequency in 13 patients with chronic migraine [64]. In a proof-of-concept open label study by Vigano’ et al, anodal current was applied over the visual cortex in 10 episodic migraine patients, with a significant reduction of 38% in migraine frequency respect to baseline [65]. An ongoing RCT will hopefully confirm these promising results for anodal tDCS applied overt the visual cortex (NCT02122757).

### Percutaneous Mastoid Stimulation

The percutaneous mastoid stimulation (PMES) device administers an electric current through the skin behind the ear and acts by inducing fastigial nucleus stimulation in the cerebellum. Its neuroprotective effect has been previously trialed in stroke medicine [66] and more recently the device was used for migraine prevention in a randomized, double-blind, sham-controlled trial [67]. The study showed a significant reduction in migraine days (71.3%) in the treatment group, as well as a significantly higher 50% response rate, although the actual blinding degree in this study is difficult to assess. If repeated, this could be a promising new treatment modality but more evidence is needed.

### Non-Painful Brachial Electric Stimulation

Yarnitsky et al. [68] studied the effects of electrical cutaneous stimulation on the arm as an acute treatment for migraine. Seventy-one patients treated at least one attack in this prospective, randomized, double-blind, sham controlled, crossover trial. Electrical stimulation was controlled by the patient’s smartphone and delivered by electrodes mounted on an armband. Various pulse widths were used but the best clinical effect was found with the 200us stimulus. A relatively modest therapeutic gain of 24% for pain freedom at 2 h was observed over sham stimulation. Active stimulation was rated as either painful or unpleasant by 39% of participants vs. 14% for sham. Compared to other stimulator devices this device has the added advantage of being discrete—it can potentially be hidden under clothing and controlled remotely. This result needs to be replicated but remote brachial stimulation does show promise as an acute treatment.

## Invasive Neuromodulation

### Occipital Nerve Stimulation (ONS)

Dural and cervical sensory afferent neurons converge upon common second order neurons in the trigeminocervocal complex. Electrical stimulation of the greater occipital nerve may modulate central pain transmission [69, 70].

A total of three RCTs have been performed in chronic migraine. All studied ONS for a 12-week double blind phase, followed by open label phases. These lasted between 1 and 3 years depending on the study. The PRISM trial was published only in abstract form and failed to show a significant improvement in migraine frequency after ONS treatment [71]. The ONSTIM study, performed on 75 subjects, compared ONS to sham stimulation and medication management [72]. Results showed a higher responder rate of 39% (defined as the percentage of subjects who achieved a 50% or greater reduction in number of headache days per month or a three-point or greater reduction in average overall pain intensity compared with baseline) in the active adjustable stimulation group, compared to 6% of the preset stimulation group and 0% of the medically managed group. There was a high frequency of side effects, most commonly lead migration. Finally, a large RCT on 157 patients failed to show a significant difference in 50% responder rate (defined as a patient with a reduction from baseline of 50% or greater together with no increase in average headache duration) in the active ONS group, even though the 30% headache pain reduction was significantly higher with treatment [73]. At the moment, an ongoing randomized controlled trial aims at comparing the effects of ONS associated with medical treatment to sham stimulation in migraine prevention (OPTIMIZE trial - NCT01775735).

Overall, what emerges from these trials is that the effect of ONS in migraine prevention is at best modest or not significant. It is possible to argue that for the most severe and refractory cases, even mild to modest reductions in headache frequency and pain rates might still prove clinically important. This may be especially so in the limited number of individuals for whom improvement lasts for years [74]. However adverse events—mostly pain, infections and lead migration—are quite common and this, coupled with the high cost, means that widespread use of the technique is not realistic.

### Sphenopalatine Ganglion Stimulation (SNS)

The sphenopalatine ganglion (SPG) forms part of the parasympathetic outflow of the cranial trigeminal-autonomic reflex. Activation of this reflex arc is responsible for the autonomic manifestations seen in trigeminal autonomic cephalalgias [75] and it also plays a role in the pathophysiology of migraine [76]. Application of lidocaine to the SPG can abort an attack of migraine [77]. The effect of electrical SPG stimulation was therefore tested as acute migraine therapy in 11 patients [78]. Unfortunately, this study had unsatisfactory results but a randomized controlled trial is currently underway to evaluate the effect of an implanted SPG stimulator in chronic migraine (Pathway M-1 study - NCT01540799).

### High Cervical Spinal Cord Stimulation

Cervical spine stimulation delivers an electrical current to the trigeminocervical complex through the use of implantable leads. This technique was initially trialed in refractory chronic migraine in a single center open label retrospective study on 17 patients [79]. Results showed a significant reduction in both pain intensity—of more than 50% in at least two thirds of patients—and median number of migraine days—from 28 to 9. Less than 20% of subjects had major adverse events, mostly lead migration and infection. More recently, 17 individuals with refractory chronic migraine and medication overuse took part in an open-label study evaluating the efficacy of high-frequency (10 Hz) stimulation of the cervical spinal cord [80]. Results were quite promising, with 8 of the 14 patients still implanted at 6 months experiencing a > 30% reduction in headache days and 6 a > 50% reduction; these rates are higher than what is usually achieved by sham stimulation in neuromodulation RCTs. An advantage with the high stimulation frequency of this particular device is that it avoids the paresthesias caused by other implantable neurostimulators. However randomized placebo controlled trials will be required before any firm recommendations can be made for this treatment.

## Conclusions

The use of non-pharmacological treatments for migraine represents an expanding clinical practice and interesting area of research. Whenever confronted with headache patients with a complex disease or who are unwilling to accept possible drug-induced adverse events, clinicians should consider this rapidly growing armamentarium of treatment strategies and choose from the different options on the base of the desired clinical indications as well as availability.

Non-invasive neuromodulation constitutes a valuable approach with strong backing evidence, particularly in the case of sTMS and transcutaneous cranial nerve stimulation. It is however available only in specialized centers, of several, but not most, countries. Even if blinding is extremely difficult to achieve in these studies, it will be interesting to observe the future developments of ongoing trials, which hopefully will confirm the initial positive results.

Nutraceuticals, especially riboflavin and magnesium, represent a good option for patients with comorbidities and who are taking concomitant medications, or in patients who cannot tolerate drug side effects. They are also cheap and easily available.

Behavioral treatment approaches can be considered as an add-on to ongoing treatments and often offer a positive clinical improvement. They are not as available as nutraceuticals, but they can be obtained in primary and secondary care settings.

Implantable neuromodulation devices, on the other hand, should only be tentatively considered for the most serious and refractory patients who have failed multiple preventive attempts.

Large clinical trials are especially needed in this field and will hopefully allow a better understanding of headache disorders, as well as a more individual-based approach to treatment in the future.

## Electronic supplementary material

ESM 1 (PDF 711 kb)
